# *Cardinium* Localization During Its Parasitoid Wasp Host’s Development Provides Insights Into Cytoplasmic Incompatibility

**DOI:** 10.3389/fmicb.2020.606399

**Published:** 2020-12-10

**Authors:** Matthew R. Doremus, Corinne M. Stouthamer, Suzanne E. Kelly, Stephan Schmitz-Esser, Martha S. Hunter

**Affiliations:** ^1^Graduate Interdisciplinary Program in Entomology and Insect Science, University of Arizona, Tucson, AZ, United States; ^2^Department of Entomology, University of Arizona, Tucson, AZ, United States; ^3^Department of Animal Science, Iowa State University, Ames, IA, United States

**Keywords:** symbiosis, cytoplasmic incompatibility, spermatogenesis, cardinium, wolbachia, encarsia, parasitoid

## Abstract

Arthropods harbor heritable intracellular symbionts that may manipulate host reproduction to favor symbiont transmission. In cytoplasmic incompatibility (CI), the symbiont sabotages the reproduction of infected males such that high levels of offspring mortality result when they mate with uninfected females. In crosses with infected males and infected females, however (the “rescue” cross), normal numbers of offspring are produced. A common CI-inducing symbiont, *Cardinium hertigii*, causes variable levels of CI mortality in the parasitoid wasp, *Encarsia suzannae.* Previous work correlated CI-induced mortality with male development time in this system, although the timing of *Cardinium* CI-induction and the relationship between development time and CI mortality was not well understood. Here, using a combination of crosses, manipulation of development time, and fluorescence microscopy, we identify the localization and the timing of the CI-induction step in the *Cardinium-E. suzannae* system. Antibiotic treatment of adult *Cardinium*-infected males did not reduce the mortality associated with the CI phenotype, suggesting that CI-alteration occurs prior to adulthood. Our results suggest that the alteration step occurs during the pupal period, and is limited by the duration of pupal development: 1) *Encarsia* produces most sperm prior to adulthood, 2) FISH localization of *Cardinium* in testes showed an association with sperm nuclei throughout spermatogenesis but not with mature sperm, and 3) two methods of prolonging the pupal period (cool temperatures and the juvenile hormone analog methoprene) both caused greater CI mortality, suggesting the degree of alteration is limited by the duration of the pupal stage. Based on these results, we compare two models for potential mechanisms of *Cardinium* sperm modification in the context of what is known about analogous mechanisms of *Wolbachia*, a more extensively studied CI-inducing symbiont.

## Introduction

Terrestrial arthropods commonly harbor heritable intracellular bacteria that spread vertically in egg cytoplasm from infected mothers to offspring (Moran et al., 2008). As these heritable symbionts rely on female host reproduction for their own success, many manipulate host reproduction to favor infected female progeny capable of transmitting the symbiont, often at the expense of male or uninfected hosts (Doremus and Hunter, 2020). A common manipulation by symbionts is cytoplasmic incompatibility (CI), a mating incompatibility that arises between symbiont-infected males and uninfected females. The offspring of this cross do not harbor the symbiont, and die early in development. Offspring survive if the female is also infected with the same symbiont strain (O’Neill et al., 1997; Serbus et al., 2008; Werren et al., 2008; LePage and Bordenstein, 2013; Taylor et al., 2018; Chen et al., 2020; Shropshire et al., 2020). CI acts to drive the symbiont infection through a host population by granting infected females a fitness advantage relative to uninfected females (Turelli, 1994).

While some details of the molecular mechanism by which symbionts induce CI remain unclear, research on the most widespread manipulative symbiont, *Wolbachia* (in the *Alphaproteobacteria*), led to the development of the modification/rescue model (Breeuwer and Werren, 1990; Hurst, 1991; Werren, 1997). In this model, *Wolbachia* produces a *modification* factor in male gametes that alters male chromatin processing after egg fertilization in early embryogenesis. This potentially fatal alteration can be reversed by the action of a *rescue* factor produced by *Wolbachia* infecting the egg cytoplasm (Werren, 1997). Recently, two *Wolbachia* prophage genes, *cifA* and *cifB*, have been identified as rescue and modification factors, although the timing and mode of action for modification/rescue are still unclear (Beckmann et al., 2017, 2019a; LePage et al., 2017; Shropshire et al., 2018). Cytological studies looking at localization of CI-inducing *Wolbachia* found this symbiont often infects developing spermatocyte cells (Clark et al., 2002, 2003, 2008; Veneti et al., 2003). However, resident *Wolbachia* are lost from the developing sperm cell during spermiogenesis, the final stage of sperm development (Clark et al., 2002, 2003, 2008). Along with other important changes, the sperm cell elongates during spermiogenesis and most of the cytoplasmic content, including any resident *Wolbachia*, is lost (Fuller, 1993; Clark et al., 2002, 2003, 2008; Ferree et al., 2019). As *Wolbachia* is lost during this process and absent from mature sperm, the modification factor is hypothesized to be either deposited into sperm cells prior to *Wolbachia* removal (Clark et al., 2003; Chen et al., 2020) or to act upon sperm chromatin prior to sperm maturation and fertilization (Poinsot et al., 2003; Shropshire et al., 2020).

Since the discovery of *Wolbachia* CI (Yen and Barr, 1974), at least four other arthropod symbionts have also been found to induce CI, two described within the last year (Hunter et al., 2003; Takano et al., 2017; Konig et al., 2019; Rosenwald et al., 2020). However, the localization of CI symbionts during spermatogenesis has only been described for a few *Wolbachia*-insect symbioses, including drosophilid flies and the chalcidoid parasitic wasp *Nasonia vitripennis* (Clark et al., 2002, 2003, 2008). Whether other CI-inducing symbionts use sperm as a means of transferring the fatal CI factor into embryos, or take advantage of some other avenue such as seminal fluid, has not been investigated. The best studied of these other taxa is *Cardinium hertigii* (*Bacteroidetes*), which infects ∼10% of arthropod species (Russell et al., 2012; Weinert et al., 2015). *Cardinium* has been confirmed to cause CI in a diverse group of hosts including mites (Gotoh et al., 2007; Wu and Hoy, 2012), thrips (Nguyen et al., 2017), planthoppers (Nakamura et al., 2012), and chalcidoid parasitic wasps in the genus *Encarsia* (Hunter et al., 2003; White et al., 2009). Genomic analysis of a *Cardinium* strain that infects the whitefly parasitoid *Encarsia suzannae* (Hymenoptera: Aphelinidae), revealed little homology to the distantly related *Wolbachia* aside from general bacterial housekeeping genes (Penz et al., 2012; Mann et al., 2017). Notably, the two CI factors responsible for *Wolbachia* CI (*cifA, cifB*) are absent from this *Cardinium* genome, indicating an independent origin of *Cardinium* CI (Mann et al., 2017; Lindsey et al., 2018). Remarkably, despite using different genes to cause CI, the cytological defects attributed to this manipulation, most notably aberrant chromosome condensation and chromatin bridging during early mitotic events, are strikingly similar for both *Cardinium* and *Wolbachia* (Gebiola et al., 2017).

In *E. suzannae*, *Cardinium* CI causes a variable level of offspring mortality (Supplementary Figure 1). This variation in mortality may arise from variable degrees of cellular defects in CI-affected embryos (Gebiola et al., 2017). Some embryos exhibit severe defects that cause mortality within the first several cycles of embryonic mitosis, while other embryos show only mild, apparently non-lethal defects (Gebiola et al., 2017). The cause of the initial variation in CI-induced cellular defects is unclear, although we recently found stage-specific effects of temperature on the severity of *Cardinium* CI in *Encarsia suzannae*. Male pupae exposed to cool temperatures demonstrated increased CI-associated offspring mortality, while the level of CI mortality induced by cooled adult males was unaffected (Doremus et al., 2019). The cause of the stronger symbiont phenotype in males that were cooled as pupae did not result from increased symbiont density, and instead appeared to involve prolonged pupal development. Longer juvenile development at a benign temperature had already been correlated with stronger levels of CI in this system (Perlman et al., 2014). Why prolonged pupal development generates stronger CI is unclear. However, lengthening the pupal stage might increase *Cardinium* CI mortality levels if the modification step of *Cardinium* CI occurs during a specific developmental window, and spermatogenesis in this wasp occurs during the pupal stage. The duration of the period of active spermatogenesis is important for *Wolbachia* CI mortality in another parasitoid wasp, *N. vitripennis* (Bordenstein and Bordenstein, 2011). *Encarsia* spermatogenesis has not been characterized, but many parasitoid wasps in the superfamily Chalcidoidea, including *N. vitripennis*, are prospermatogenic and produce the vast majority of their lifetime sperm prior to adulthood (Gordh and Debach, 1976; Boivin et al., 2005; Ferree et al., 2019).

Here we establish a framework for *Cardinium* CI modification by investigating the timing of the modification step of *Cardinium* CI and testing if ideas developed for *Wolbachia* CI in various insect hosts apply to the independently evolved *Cardinium* CI system in the wasp *E. suzannae*. Specifically, we test if *Cardinium* modification occurs prior to the wasp adult stage, and whether *Cardinium* associates with sperm cells during *Encarsia* spermatogenesis. Support for modification occurring during the wasp pupal stage would be provided by demonstration that a) an active *Cardinium* infection of adult males is not required to cause CI because modification is completed before adulthood, b) *E. suzannae* produces most of its mature sperm during pupation, and c) *Cardinium* cells localize within developing spermatocytes, and are lost as sperm cells approach maturity, like *Wolbachia* (Clark et al., 2002, 2003, 2008). To test this hypothesis, we used antibiotic treatment of infected adult males to test whether an active infection is required for the *Cardinium* CI phenotype, and used fluorescence microscopy to characterize both *Encarsia* spermatogenesis and *Cardinium* localization during this developmental process. In agreement with the hypothesis that modification occurs during the pupal stage prior to adulthood, we found that antibiotic treatment of adult males did not diminish the CI phenotype. We also found that *Cardinium* co-localizes with developing sperm cells during spermatogenesis and is then removed in the final stages of sperm development. Lastly, we found that experimentally prolonging the duration of the male pupal stage, with either cool temperatures or the juvenile hormone (JH) analog methoprene, increases offspring mortality associated with CI, suggesting that sperm modification is time-limited and occurs during the pupal stage.

## Materials and Methods

### Insect Cultures

*Encarsia suzannae* (formerly *E. pergandiella*; Gebiola et al., 2017) is a minute parasitoid wasp (Hymenoptera: Aphelinidae) with an unusual life history; females develop within immature whitefly nymphs, while males develop as hyperparasitoids, meaning they are parasitic on conspecific pupal females or other pupal aphelinid wasps (Hunter and Woolley, 2001). For the current study, female wasp progeny were produced by providing mated females with *Bemisia tabaci* (sweet potato whitefly) nymphs. Haploid male wasps were produced separately by providing virgin female *E. suzannae* with pupae of a second aphelinid species, *Eretmocerus emiratus*. Wasps were maintained at 27°C unless otherwise noted.

### CI Crosses

*Encarsia suzannae* is naturally infected with the CI-inducing *c*Eper1 strain of *Cardinium hertigii* (Hunter et al., 2003; Penz et al., 2012; Perlman et al., 2014; Doremus et al., 2019). *Cardinium*-free “cured” cultures of *E. suzannae* used in CI crosses were previously established by feeding female wasps 50 mg/ml rifampicin in honey for 48 h prior to egg laying for three consecutive generations, followed by more than 10 generations of wasp culture without antibiotic treatment (Hunter et al., 2003). The *Cardinium* strain in this study induces ∼70–80% offspring mortality in CI crosses, although CI-induced mortality is variable (Supplementary Figure 1) (Perlman et al., 2014; Doremus et al., 2019).

To test for the degree of CI mortality, crossing assays were performed. Male wasps were monitored for adult emergence and provided with honey upon emergence. Two days later, males were mated to a virgin female 1–6 days old: either a *Cardinium*-infected female (expected to be a viable “rescue” cross) to test for male fertility, or to an uninfected female (expected to be a CI cross with high levels of mortality). Upon observation of successful mating, individual females were moved to a parasitism arena consisting of a disk of cowpea leaf (*Vigna unguiculata*) with ∼50–70 2nd–3rd instar whitefly nymphs on 1% agar (in DI water) in a 35 mm Petri dish with a screened lid (Hunter et al., 2003). Females were left to parasitize whitefly hosts for 24 h before removal. Parasitism by *E. suzannae* arrests whitefly development in the 4th instar, regardless of whether the wasp egg or larva survives (Hunter et al., 2003). Ten days after parasitism we counted the number of surviving wasp pupae and arrested whiteflies (the latter a proxy for CI-induced wasp mortality). CI-induced offspring mortality was compared across treatments for rescue and CI crosses using logistic regression with a quasibinomial distribution in R 3.3.1 (R Core Team, 2016).

### Effects of Adult Antibiotic Treatment on *Cardinium* CI Strength

To test if an active *Cardinium* infection in adult male wasps is required for CI-induced offspring mortality, we fed adult male *E. suzannae* with honey containing 50 mg/ml of rifampicin, a concentration of rifampicin that consistently cures *Cardinium* infections (Hunter et al., 2003). Males were fed either antibiotic-treated honey or plain honey for 7 days, at which point they were allowed to mate with either an infected female (viable rescue cross) to test for effects of antibiotic treatment on male fertility, or an uninfected female (inviable CI cross) to test for effects of antibiotic treatment on *Cardinium*-induced CI. After mating, females were placed in parasitism arenas and males were stored at −80°C. To confirm that antibiotic treatment resulted in *Cardinium* loss, we extracted DNA from treated males and estimated *Cardinium* density using quantitative PCR (qPCR) using primers and reaction parameters described previously (White et al., 2009; Doremus et al., 2019). See SI for additional methods details.

### Fluorescence *in situ* Hybridization and Immunofluorescence

To investigate *E. suzannae* spermatogenesis, we stained testes with DAPI, a nuclear stain, and to determine *Cardinium* localization within developing *E. suzannae* testes, we performed Fluorescence *in situ* hybridization (FISH) using Cy3 double-labeled probes for *Cardinium* 16S rRNA (Ch1162 5′- (Cy3)-TTGACCTCATCCTCTCCT (Cy3-Q) −3′). Male wasps raised at 27°C were collected at developmental stages corresponding to easily distinguishable morphological markers of their progression through pupation. These stages included white pupae with little to no eye pigmentation (“white”), pupae with red-pigmented eyes (“red-eyed”), fully melanized pupae (“black”), and 2-day old adults (Figures 3A–6A). Males were stored at −80°C until dissection.

Using a FISH protocol modified from Daims et al. (2004), testes were removed from males in 1× PBS on a coverslip and promptly fixed in 20 μL of 4% paraformaldehyde for 20 min at room temperature. The fixative was then removed, and the samples were washed 2–3 times with ddH_2_O. Samples then underwent an ethanol dehydration series of 3 min rinses in 50, 80, and 96% ethanol (in ddH_2_O). Before hybridization, testes were attached to the coverslip by covering them with a thin layer of 0.5% agarose (pulse field gel electrophoresis grade) gel (diH_2_O). Samples were then incubated with 10 μL hybridization buffer [10% formamide, 0.9 M NaCL, 20 mM Tris HCl (pH 8), 0.01% SDS] and 1 μL cy3 probes (30 ng/μL) in the dark at 46°C for ∼2 h. After hybridization, samples were washed in washing buffer [10% formamide, 0.45 M NaCl, 20 mM Tris HCl (ph 8)] for ∼10 min in a 48°C water bath. Samples were then placed in ice cold ddH_2_O for 1–2 seconds and dried with compressed air. They were then either immediately stained with 5 μL DAPI (10 μg/mL in TBST) for 5 min or kept at −20°C in the dark until DAPI staining. Coverslips were mounted with mounting media [80% glycerol, 20% TBST with 2% *n*-propyl-gallate (Sigma)]. To test the specificity of the *Cardinium* 16S rRNA probe, we also performed FISH on testes from males that were cured of *Cardinium* (Supplementary Figure 2). Images were collected as z-stacks using a Zeiss LSM 880 inverted confocal microscope using a 63x lens with oil immersion. DAPI and cy3 fluorescence were captured using a 405 and 561 nm excitation beam, respectively. Here we present representative images that capture the center depth of the testis. Relative fluorescence intensity for *Cardinium* across the apical, middle, and basal portions of testes were calculated using the Zen Blue Software (Zeiss). Testes from at least six males of each stage were prepared and imaged for comparisons. Due to the variability in FISH performance across slides and assays, we compared fluorescence intensity only within testes on the same slide.

### Effects of Early and Late Pupal Cold Exposure on *Cardinium* CI

To test whether CI strength is influenced when males experience cold in either early or late stages of pupal development, we exposed male pupae to one of two cool temperature treatments. The first exposure consisted of development at 20°C day/17°C night from the onset of the prepupal stage until the first signs of melanization, which included the white and red-eyed pupal stages. During this developmental period, most developing sperm cysts are undergoing multiple mitotic divisions, culminating with the onset of spermiogenesis (sperm elongation) in several cysts. The second exposure consisted of development at 20°C day/17°C night during the black pupal stage, from the onset of cuticular melanization until 2 days post-adult emergence when males from either treatment were allowed to mate with uninfected females (CI cross). The testis is markedly different during this second half of pupation and early adulthood, with most cysts undergoing and completing spermiogenesis, during which time *Cardinium* is lost from sperm cells. After temperature exposure, we mated males from the two cold treatments as well as control males raised at 27°C to uninfected females. We then placed these females in parasitism arenas and assessed their offspring survival.

### Cold and Methoprene Exposure Effects on Male Pupal Development

To test whether prolonged pupal development, independent of cool temperatures, is responsible for stronger CI in the *Cardinium-Encarsia* system, we exposed 3rd instar male *E. suzannae* larvae to methoprene, an analog to JH that plays multiple roles in insect development (Jindra et al., 2013). During the 2nd instar, the male *E. suzannae* larva emerges through the cuticle of its wasp host (*Er. emiratus*) and proceeds to consume the *Er. emiratus* as an ectoparasitoid residing within the whitefly cuticle (Gerling, 1966). To topically expose a male larva to methoprene, we first created a small hole in the whitefly cuticle surrounding the larva using fine minuten insect pins (Bioquip Co). Next, using a microinjector (Drummond Nanoject III) fitted with a borosilicate glass needle, we topically delivered ∼10 nL of methoprene (0.1 mg/mL in acetone; Santa Cruz Biotechnology) or an acetone control through the small hole in the whitefly cuticle onto the visible 3rd instar larva. The dose was determined empirically; higher concentrations of methoprene resulted in complete *E. suzannae* mortality. After exposure, individual larvae were moved to 1.2 mL vials in a Percival incubator at 27°C and monitored daily until adult emergence. We also included a cool temperature treatment for the same cohort to compare with the methoprene treatment. Pupal development time was recorded for males in each of the treatments (27°C control, 20–17°C cold, acetone or methoprene exposure; *n* = 37–48). Development time was analyzed across treatments using multiple pairwise *T*-tests with the *p*-values adjusted by the Benjamini-Hochberg correction for multiple comparisons R 3.3.1 (R Core Team, 2016). After adult emergence, males were mated with either an infected (rescue cross) or uninfected female (CI cross) and offspring survival was assessed.

## Results

### Adult Exposure to Antibiotics Does Not Modify CI Strength

**FIGURE 1:**
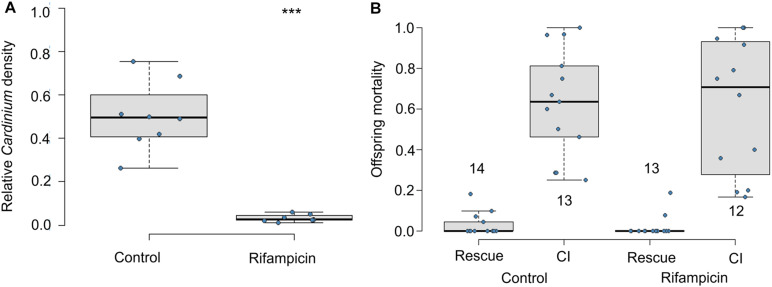
Effects of adult antibiotic (rifampicin) exposure on **(A)** relative density of *Cardinium* in male wasps determined by qPCR (*Cardinium gyrB* copy number/host *ef1A* copy number), and **(B)** resulting CI-induced offspring mortality. Sample size in A = 8 samples with three technical replicates. Sample sizes for **(B)** are given above or below whisker plots. Statistical comparisons between honey-fed control and rifampicin-treated adults were determined by Mann-Whitney *U*-test in **(A)** and, in **(B)**, by quasibinomial logistic regression between equivalent cross types in rifampicin and control treatments. Asterisks indicate statistical significance. ***p < 0.001.

If living *Cardinium* in adult males is required for CI, offspring of antibiotic-fed adult males should not die of CI. Exposure to antibiotics significantly reduced the relative density of *Cardinium* in male wasps (Figure 1A; Mann Whitney *U*-test *p* = 0.00016). However, consistent with CI modification occurring during the male host pupal period, offspring of antibiotic-treated adult males showed similar levels of CI mortality as did the honey-fed controls (Figure 1B; *F*_1_,_23_ = 0.09, *p* = 0.75). Antibiotics also did not affect male fertility; antibiotic-treated males in the rescue cross showed negligible levels of offspring mortality, comparable to the fertility of honey-fed control males (Figure 1B; *F*_1_,_25_ = 0.4, *p* = 0.5).

### Cardinium Within-Testis Localization During Host Spermatogenesis

**FIGURE 2:**
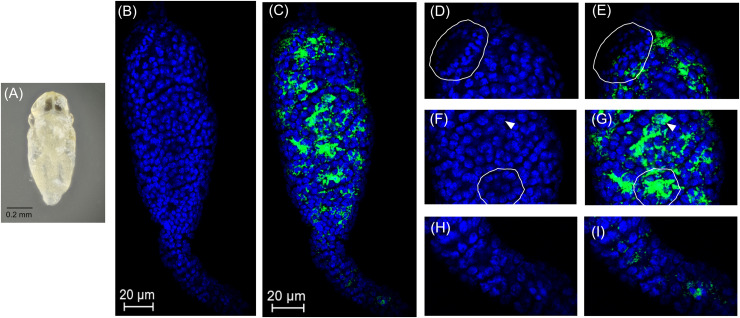
*Cardinium* localization in “white” pupal testes. The distal portion of the testis is at the top of all panels. **(A)** White *E. suzannae* male pupa. **(B)** Entire pupal testis showing DAPI-stained host nuclei (blue) and **(C)** also showing cy3-stained *Cardinium* 16S rRNA (green). **(D)** Magnified distal region, showing spermatocytes and **(E)** *Cardinium* associations with host nuclei in the same region. The circled region shows a cyst with condensed nuclei beginning to align in the cup formation representing entry into spermiogenesis. **(F)** Magnified mid-region of testis, showing interphase spermatocyte nuclei and **(G)** *Cardinium* relative to host nuclei. The arrowhead in F and G refers to an interphase nucleus engulfed by *Cardinium* cells and the circled region shows nuclei associated with a dense patch of *Cardinium* cells. **(H)** Magnified apical region of testis. **(I)** Location of *Cardinium* in apical region of testis, which contains fewer *Cardinium* cells relative to the rest of the testis.

**FIGURE 3:**
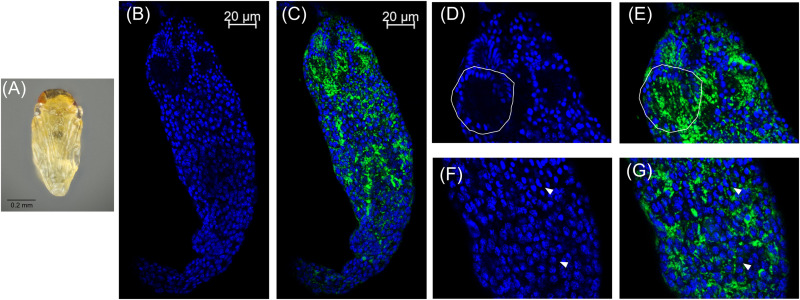
*Cardinium* localization in “red-eyed” pupal testes. **(A)** Red-eyed *E. suzannae* male pupa. **(B)** Entire red-eye pupal testis oriented from top (distal) – down (apical) showing DAPI-stained host nuclei (blue) and **(C)** cy3-stained *Cardinium* 16S rRNA (green) with host nuclei. **(D)** Magnified distal region of testis, with DAPI-stained nuclei and **(E)** cy3-stained *Cardinium* 16S rRNA (green) with host nuclei. Solid circles show a cyst with elongating nuclei forming the characteristic c-shape. What appears to be empty space in these cysts is occupied by the cytoplasm of the elongating sperm tail. **(F)** Magnified mid-region of testis showing DAPI-stained host nuclei (blue) and **(G)** cy3-stained *Cardinium* 16S rRNA (green) with host nuclei. Here the *Cardinium* cells appear more evenly distributed across cysts than in the white pupal stage. Arrowheads show representative individual nuclei that have not begun elongating with closely associating *Cardinium* cells.

**FIGURE 4:**
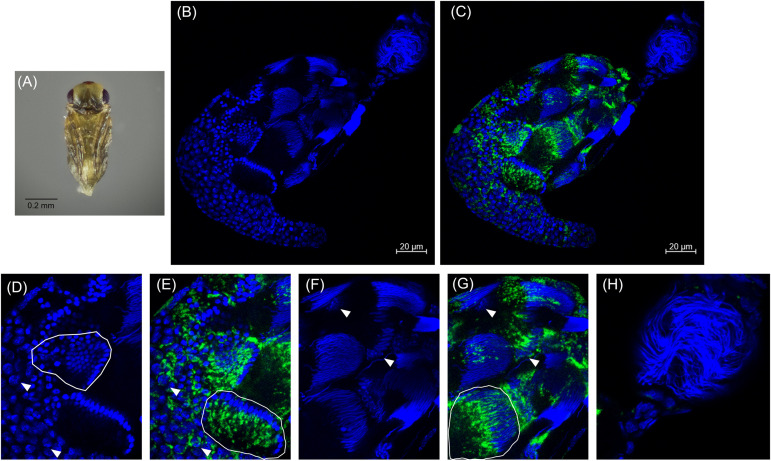
*Cardinium* localization in “black” pupal testes. **(A)** Black *E. suzannae* male pupa. **(B)** Entire black pupal testis oriented from lower left (apical) to upper right (distal) showing DAPI-stained host nuclei (blue) and **(C)** cy3-stained *Cardinium* 16S rRNA (green) with host nuclei. **(D)** Magnified mid-region showing DAPI-stained host nuclei (blue) and **(E)** cy3-stained *Cardinium* 16S rRNA (green) with host nuclei. Circled regions show cysts with condensed nuclei forming the cup formation indicative of entry into spermiogenesis. Arrowheads show individual interphase nuclei with closely associating *Cardinium*. **(F)** Magnified distal region of testis with DAPI-stained nuclei and **(G)** cy3-stained *Cardinium* 16S rRNA (green) with host nuclei. The circled region in **(G)** shows a cyst with elongating nuclei that appears to be losing its resident *Cardinium*. Empty space in these cysts is occupied by the cytoplasm of the elongating sperm tail. Arrowheads show the larger nucleus of either a somatic cyst cell or a trophocyte (nutritional cells). **(H)** Magnified view of the seminal vesicle containing mature sperm. No *Cardinium* cells are evident.

**FIGURE 5:**
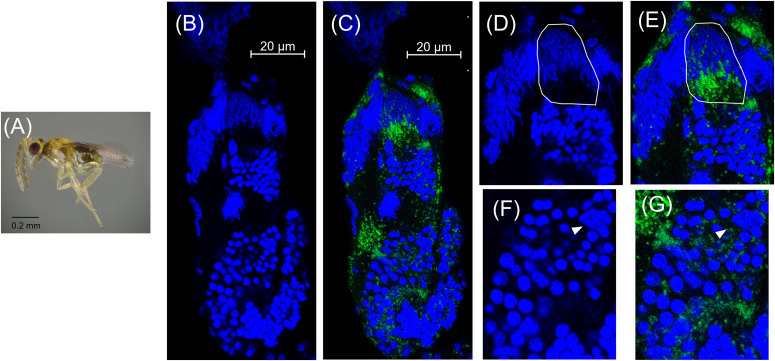
*Cardinium* localization in adult testes two days post-emergence. **(A)** Adult male *E. suzannae*. **(B)** Entire adult testis oriented from top (distal) to bottom (apical) with DAPI-stained host nuclei only (blue) and **(C)** DAPI + cy3-stained *Cardinium* cells (green). **(D)** Magnified distal region of testis with DAPI-stained nuclei and **(E)** cy3-stained *Cardinium* with host nuclei. Circled region shows a cyst in spermiogenesis with elongating nuclei. Empty space in these cysts is occupied by the cytoplasm of the elongating sperm tail. **(F)** Magnified mid-region showing DAPI-stained host nuclei and **(G)** cy3-stained *Cardinium* with host nuclei. Arrowheads show the larger nucleus of a somatic cell.

Spermatogenesis has not been characterized in *Encarsia* species. We found spermatogenesis in *E. suzannae* to be similar to that of *N. vitripennis*, another chalcidoid parasitic wasp, documented by Ferree et al. (2019). As in *N. vitripennis*, the *E. suzannae* testis consists of a single ellipsoidal follicle. Unlike the *N. vitripennis* testis however, the apical portion of the *E. suzannae* testis is much narrower than the distal portion where the developing sperm cells are located (Figures 2B,H). The cells in this apical region do not appear to develop like the sperm cells occupying the downstream region of the testis (Figures 2B,H, 3B, 4B, 5B). It is possible that this region houses the cellular hub that gives rise to spermatagonia (Toomey and Frydman, 2014), or alternatively may consist of somatic cells that perform a supportive function, like trophocytes present in other wasp species (Ferree et al., 2019). Interestingly, *Cardinium* density is significantly reduced in this apical region relative to the larger, distal portion of the testis that houses actively developing sperm cells (Figures 2E,G,I and Supplementary Figure 3).

In early stage, “white” pupae, we found spermatocyte nuclei throughout the testis, indicating that initial mitotic divisions occur prior to the pupal stage (Figures 2A,B,D,F). The spermatocytes appear to be interconnected during this stage, as individualization of sperm cells generally occurs during spermiogenesis, the elongation process of sperm development. *Encarsia* sperm cells are clearly organized in bundles or cysts (Fuller, 1993; Ferree et al., 2019). These cysts of developing sperm make up almost the entirety of the larger, distal region of the testis at this stage (Figures 2B,D,F). We observed *Cardinium* scattered throughout the distal region of the white pupal testis, often close to spermatocyte nuclei (Figures 2C,E,G). The density of *Cardinium* varied substantially around nuclei, with high densities of symbionts in some areas, where the nucleus appeared nearly engulfed by solid bright patches of *Cardinium* signal. Other regions showed apparently fewer symbiont cells (Figures 2C,E,G). Unfortunately, given the minute size and frailty of *E. suzannae* testes (∼8× smaller than *N. vitripennis* testes) (Ferree et al., 2019), we were unable to dissect intact individual cysts to quantify resident *Cardinium* density in individual cysts.

In these white stage pupae, we also observed the most distal spermatocyte nuclei aligning in a hemispherical “cup” shape indicative of entry into spermiogenesis (Figures 2B,D) (Ferree et al., 2019). The nuclei of these cysts were trailed by extending cytoplasm that contained *Cardinium* cells (Figure 2C). Approximately 24 h later, in the testes of “red-eyed” pupae, more cysts had aligned in the cup formation and initiated spermiogenesis (Figures 3A,B,D). *Cardinium* cells were still present in the most distal elongating sperm cells (Figures 3C,E), but these bacterial cells were trailing the aligning nuclei along with the extending cytoplasm (Figure 3E). *Cardinium* cells also appeared more uniformly distributed among nuclei compared to the earlier white pupal stage (Figures 2G, 3C,F,G). In this stage *Cardinium* appeared in close proximity to most spermatocyte nuclei (Figures 3C,F,G).

Approximately 48 h after the red-eyed stage, the pupal cuticle darkens to the “black” pupal stage. Here we observed that most of the developing sperm cells had progressed into spermiogenesis (Figures 4A,B,F and Supplementary Figure 4). The earliest developing cysts had fully matured and the now individualized sperm had migrated out of the testis into the seminal vesicle, the storage organ that houses sperm until mating (Figures 4B,H). The black stage is the first pupal stage in which we observed mature sperm within the seminal vesicle (Figure 4B; Supplementary Figures 4, 5). Within the testis, we observed elongating voids that trailed maturing sperm nuclei, indicative of the growing sperm tails composed primarily of α-tubulin (Supplementary Figure 6). Sperm at this stage individualize and lose most of their cytoplasmic contents. The nuclei also become hyper-condensed and elongate as histones are removed and the DNA is wrapped around sperm proteins, including protamines (Ferree et al., 2019). *Cardinium* trailed behind these elongated nuclei, suggesting that the symbiont is lost from spermatids during the cytoplasm removal process of spermiogenesis (Figures 4C,G) (Clark et al., 2002, 2003, 2008). We did not observe *Cardinium* in the seminal vesicle (Figures 4B,H and Supplementary Figure 5), nor did we find *Cardinium* within or associated with individual sperm cells after we removed sperm from the seminal vesicle (Figure 4H; Supplementary Figure 6). Most *Cardinium* were within the testis itself, although we also found a small number of *Cardinium* cells in the cytoplasm of some epithelial cells of the downstream reproductive tract (Figures 4C,H and Supplementary Figure 5).

In adult wasps that emerged ∼24 h after the onset of the black pupal stage, the testes were superficially similar in appearance to those of the previous stage (Figures 4B, 5A,B). However, there were fewer spermatocyte nuclei in the adult testis, and elongating sperm cysts were not as tightly packed together (Figures 5B,D). Many of the elongating spermatids that were observed in the black pupal stage were missing in the adult stage, likely because they had completed maturation and migrated to the seminal vesicles (Figure 5B). Approximately 2–5 cysts of nearly mature sperm cells remained in the testes of 2-day-old adults; however, the testes of 4-day-old unmated males contained a similar number of unmigrated spermatid bundles within the testis (Figures 5B,D and Supplementary Figure 7). There were also 2–3 cysts anterior to the mature sperm that showed condensed nuclei in the early stages of spermiogenesis, but these also did not appear to align in the cup formation in 4-day-old adult testes, suggesting that they do not develop further, at least in the absence of sperm depletion (Figures 5B,D,F and Supplementary Figure 7). Among individual adult males, *Cardinium* was present in adult testes at varying densities, consistent with the variability found among previous qPCR estimates of whole host *Cardinium* density (Figure 5C; Supplementary Figure 8) (Doremus et al., 2019). However, while the remaining 1–2 spermatocyte bundles that had yet to mature still harbored *Cardinium* (Figures 5E,G), most *Cardinium* cells were either in the process of being stripped away from the remaining spermatid nuclei or were associating with the more clearly visible, larger nuclei that likely belonged to somatic cells (Figures 5F,G).

We note that here we only examined the testes of unmated males, equivalent to males used in the CI crossing experiments described. However, in other observations, testes of males that had mated with up to 20 females appeared similar to those of virgin males, with similar numbers of cysts in earlier stages of spermatogenesis, and seminal vesicles with ample sperm stores (Supplementary Figure 7).

### Both Early and Late Pupal Cold Exposure Causes Increased CI Mortality

**FIGURE 6:**
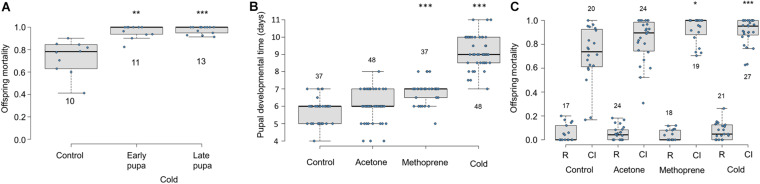
Effects of cold temperature and methoprene on *Cardinium*-induced CI and *E. suzannae* male development **(A)** Effects of early or late male pupal exposure to cool temperatures (20°C day/17°C night) on *Cardinium*-induced CI mortality of offspring. The early pupal treatment lasted from the onset of pupation, through the white and red-eyed stages until the onset of cuticular melanization. The late pupal treatment began at the onset of cuticular melanization, through the black pupal stage, and ended at adult emergence. **(B)** Effects of male wasp exposure to methoprene and cool temperatures (20°C day/17°C night) on duration of pupal development and **(C)** CI-induced offspring mortality. The numbers above or below the box and whisker plots represent biological replicates (*n* = 10–48 for all treatments). Rescue and CI crosses are denoted by R and CI, respectively, except in **(A)**, which only shows CI crosses. In **(A)** and **(C)**, logistic regression with a quasibinomial distribution was used to compare offspring mortality of the control R or CI cross with each of the comparable treatment crosses (or to the control CI cross in **A**). In **(B)**, significant differences from the control treatment were determined by multiple pairwise Student’s *T*-tests with Benjamini-Hochberg corrected p-values. Asterisks denote *P* values (*p = 0.05, **p < 0.01, and ***p < 0.001).

Exposure to cool temperatures causes pupal developmental delay and increases *Cardinium*-induced CI (Doremus et al., 2019). To test which stages of sperm development (i.e., pre-elongation or elongation phases) are most important for *Cardinium* sperm modification, we exposed early and late male pupae to cool temperatures. We found that both early and late temperature treatments resulted in increased and nearly complete CI mortality compared to control males (Figure 6A; Early *F*_1_,_32_ = 10.96, *p* < 0.001; Late *F*_1_,_31_ = 39.19, *p* < 0.0001). Early and late treatment offspring mortality did not significantly differ from each other (Figure 6A; *F*_1_,_22_ = 0.88, *p* = 0.35).

### Pupal Developmental Time and CI-Associated Mortality

To test the effect of pupal development time on *Cardinium*-induced CI independent of temperature, we exposed 3rd instar male *E. suzannae* larvae to a topical application of methoprene (in acetone), acetone, or to cool temperatures (20–17°C). We found that methoprene application significantly extended male pupal development compared to both the untreated and acetone-treated control males, resulting on average in a ∼24 h developmental delay (Figure 6B; Student *T*-test *p*^*met–con*^ < 0.0001; *p*^*met–ace*^ < 0.0001). As previously shown, exposure to cool temperatures also significantly delayed development by more than 72 h compared to the control treatment (Figure 6B; *p* < 0.0001), and by 36 h relative to methoprene exposure (Figure 6B; *p* < 0.0001). The acetone treatment did not significantly prolong pupal development compared to the untreated control (Figure 6B; *p* = 0.16).

We found that offspring of *Cardinium*-infected males raised at the 27°C control temperature suffered a median of 73% offspring mortality in CI crosses (Figure 6C) (Perlman et al., 2014; Doremus et al., 2019). Acetone exposure did not significantly increase CI strength compared to the untreated control (Figure 6C; *F*_1_,_88_ = 0.64, *p* = 0.43). Methoprene induced significantly higher offspring mortality (i.e., stronger CI phenotype) relative to untreated controls (Figure 6C; logistic regression *F*_1_,_87_ = 4.71, *p* = 0.03), as well as compared with acetone-exposed controls (Figure 6C; *F*_1_,_41_ = 4.16, *p* = 0.05), causing 100% median offspring mortality. The increased CI mortality caused by methoprene exposure was comparable to that of temperature (Figure 6C; *F*_1_,_44_ = 0.21, *p* = 0.65). As in previous trials, males exposed to cooler temperatures during the pupal stage also showed stronger CI (95% median mortality) compared to 27°C control males (Figure 6C; *F*_1_,_86_ = 13.49, *p* = 0.0004).

## Discussion

To generate a mechanistic framework for *Cardinium*-induced CI in the minute parasitic wasp *Encarsia suzannae*, we determined the timing and localization of *Cardinium* sperm modification. We found that antibiotic treatment of adult male *E. suzannae* did not interfere with the CI phenotype. This indicates that the sperm alteration that leads to the CI phenotype occurs before the adult stage and does not require an active *Cardinium* infection at the time of male mating (Figure 1).

We also found that *E. suzannae* produces most of its sperm during the pupal stage and that *Cardinium* infects developing sperm cells. In spermiogenesis, the final stage of sperm development, *Cardinium* is lost from these cells and is absent from mature sperm (Figures 2–5). Together, these results strongly suggest that *Cardinium* either alters sperm cells directly or uses sperm cells as a vehicle to deliver the fatal, unknown, CI factor into developing embryos. Further, this process of sperm modification occurs during the host pupal stage when sperm are developing.

**FIGURE 7:**
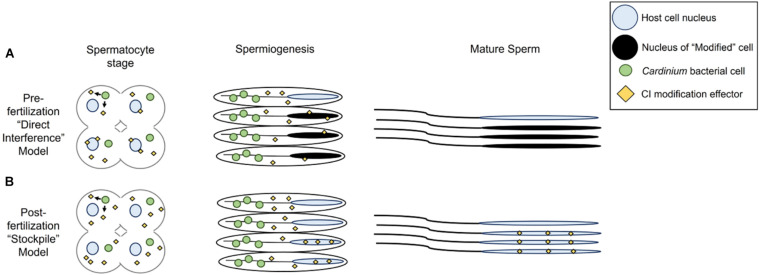
Models for *Cardinium* sperm alteration. **(A)** Pre-fertilization “host modification” model, in which *Cardinium* produces a CI effector during early spermatogenesis stages when the symbiont is close to spermatocyte nuclei. The host target, likely a protein involved in spermiogenesis-associated nuclear reorganization, becomes available for alteration during elongation or spermiogenesis. CI-altered mature sperm enter seminal vesicle. **(B)** Post-fertilization “toxin-antidote” model, in which *Cardinium* stockpiles sperm cells with the CI effector throughout spermatogenesis until symbiont removal during spermiogenesis. The CI effector is packaged into spermatids, but the final host target, potentially a protein involved in re-modeling of sperm chromatin from its elongated state, is available in the egg cytoplasm. Mature sperm must be enriched for the CI effector at some threshold to cause cellular defects in the egg and mortality.

Building on these initial results, we extended male host development time with two methods, cold and the JH analog methoprene. We previously found that cooler temperatures prolonged male wasp development and increased CI strength (Doremus et al., 2019). Here we specifically tested the importance of early and late pupal development time on CI strength by exposing these pupal stages to cooler temperatures. These two treatments corresponded with the white to red-eyed stages (“early”) and the mottled to black stages of pupal development (“late”). We found exposure to cool temperatures resulted in uniformly strong CI regardless of stage exposed (Figure 6). In later pupal stages, many sperm cells had begun losing their resident *Cardinium* (Figure 4), yet a temperature-induced delay at this stage was still able to induce strong CI. This suggests that the modification process begins in early sperm development and continues through spermiogenesis, when *Cardinium* is being removed (Figures 4, 6). Perhaps the presence of *Cardinium* within these cells is not required in this late stage for modification to proceed. The CI effector(s) may act independently at this point, either by interacting with a host target or entering the sperm nucleus (Figures 4, 6, 7).

We found that the testes of cooled male wasps showed similar densities of *Cardinium* and a similar developmental schedule of spermatogenesis compared to control wasps of the same stage (Supplementary Figure 8). However, cooled males take more than twice as long to complete their pupal stage compared to untreated males (Supplementary Figure 8), and exhibit stronger CI (Figure 6). That the JH analog methoprene also caused a developmental delay and stronger CI suggests that it is the developmental delay, and not a temperature or methoprene effect on *Cardinium* density, gene expression, or protein interactions, that is the primary cause of increased CI mortality (Figure 6) (Doremus et al., 2019). Without testing even more variables, however, we cannot rule out the possibility that temperature and methoprene influence CI independently, and the developmental delay associated with both treatments is coincidental and not the critical factor influencing CI strength (Liu et al., 2014).

The cellular defects responsible for *Cardinium* CI mortality have previously been found to vary amongst embryos, with some embryos suffering more severe defects and earlier mortality; other embryos appear to suffer only mild defects that may not be lethal (Gebiola et al., 2017). Differences in the duration that sperm cells are exposed to *Cardinium*, either among males or due to sperm cell position within individual testes, may explain some of the differences in defects arising from CI modification (Gebiola et al., 2017). Together with the results of the current study, this suggests that there is a specific developmental window from the onset of spermatogenesis to sperm maturation, during which the host sperm cell is susceptible to CI modification. Under “typical” developmental conditions, this period is too short for *Cardinium* to fully modify every sperm cell. This could be due to a threshold of effects required to render a sperm cell fatally modified. Under this scenario, we would expect to see the typically variable levels of *Cardinium* CI resulting from a mixture of sperm cells modified to various degrees, with most cells reaching a fatal level of modification, while others receive a smaller dose that the developing embryo can endure. Extending the modification window may then result in a higher proportion of sperm reaching the fatal modification threshold and uniformly strong CI. A similar threshold model has been applied to *Wolbachia*, although for *Wolbachia*, symbiont density and not developmental period has been shown to determine CI strength (Breeuwer and Werren, 1993; Hurst et al., 2000; Bordenstein and Bordenstein, 2011).

Based on the results of this study, two models developed for the *Wolbachia* modification mechanism may apply to how *Cardinium* lethally modifies host sperm (Hurst, 1991; Tram and Sullivan, 2002; Poinsot et al., 2003; Bossan et al., 2011; Beckmann et al., 2019a; Chen et al., 2020; Shropshire et al., 2020). The two models differ in whether the mature sperm cells harbor a *Cardinium*-derived CI effector (a stockpiling method, or the “toxin-antidote” or “lock-and-key” model in the language of the *Wolbachia* CI literature; Hurst, 1991; Poinsot et al., 2003; Beckmann et al., 2019a; Chen et al., 2020) or not (a direct alteration method or the “host modification” model of *Wolbachia* CI; Tram and Sullivan, 2002; Poinsot et al., 2003; Bossan et al., 2011; Shropshire et al., 2020). The models also differ in the proposed site of the CI effector-host target interaction (Figure 7) (Poinsot et al., 2003; Chen et al., 2020; Shropshire et al., 2020). In the case of direct alteration, *Cardinium* produces an effector that works directly on the host target(s) throughout sperm development, beginning in the early stages of development when sperm cells are replicating and still interconnected, and proceeding through sperm maturation (Figure 7A) (Ferree et al., 2019). Possible targets include protamines, which are small molecules responsible for remodeling the sperm nucleus into a hyper-condensed, inactive state (Raja and Renkawitz-Pohl, 2005), seminal fluid proteins (Yuan et al., 2015), or other proteins and RNA associated with reproductive success (Liu et al., 2014; Ju et al., 2017; Huang et al., 2019; Zheng et al., 2019). Target alteration could affect downstream protamine replacement with histones during fertilization. In this model, sperm are altered within the testis before fertilization and no effectors travel with the sperm (Poinsot et al., 2003; Shropshire et al., 2020). In the “toxin-antidote” model, *Cardinium* stockpiles the CI effector during sperm development (Figure 7B). The effector is then packaged within the mature sperm cell, perhaps by localizing within the nucleus during the elongation process. Upon entry of the sperm to the egg cytoplasm and prior to the start of mitosis, the CI effector then interacts with its host target (Hurst, 1991; Poinsot et al., 2003; Chen et al., 2020). Possible targets could include proteins involved in remodeling the elongated sperm nucleus via the reincorporation of maternal histones for chromatin wrapping (Loppin et al., 2005; Landmann et al., 2009; Zheng et al., 2011; Beckmann et al., 2019b). Detailed proteomic surveys of *Encarsia* seminal vesicles for *Cardinium* proteins, which contain mature sperm but not *Cardinium* (Figure 4), may provide an answer as to which model is appropriate for *Cardinium*-induced CI.

The residence of *Cardinium* within developing sperm cells is reminiscent of the localization of *Wolbachia*, particularly the parasitoid, *Nasonia*, which undergoes a similar sperm developmental schedule (Figures 2–5) (Clark et al., 2002, 2003, 2008). Both symbionts are found infecting developing sperm cells and both symbionts are removed from these cells during the final stages of sperm development, specifically during the elongation process of spermiogenesis (Figures 2–5) (Clark et al., 2002, 2003, 2008). However, *Cardinium* appears to infect a higher proportion of developing sperm cysts than *Wolbachia*, which infects an estimated 28% of cysts in *Nasonia* (Clark et al., 2008). Despite the majority of *Nasonia* sperm cysts being apparently uninfected by *Wolbachia*, CI-induced mortality is uniformly strong in this system, causing ∼97% mortality (Clark et al., 2008). This contrasts with *Cardinum*, which infects a higher proportion of cysts (∼96% of cysts) yet causes a milder CI phenotype. It is possible that *Cardinium* CI effectors do not diffuse across cyst membranes, as seems to be the case for *Wolbachia* infecting *Nasonia* testes (Clark et al., 2008). Alternatively, CI mortality caused by *Cardinium* may require a higher threshold of modification than that of *Wolbachia*, whether it is due to accumulation and packaging of CI effectors or to direct chromatin alteration (Figure 7).

That *Cardinium* and *Wolbachia*, which both sabotage host sperm to induce CI, show substantial similarities at the cellular level is intriguing. Identification of the *Cardinium* CI effectors, as well as the host targets for these effectors, may reveal mechanistic convergence of these independent CI systems. Alternatively, distinct processes might appear similar at a cellular level (Doremus and Hunter, 2020). The study of more *Cardinium*-CI symbioses, as well as recently discovered CI symbionts, will aid in generating a generalized knowledge both of how CI-inducing bacteria interact with their insect hosts during spermatogenesis and how that interaction causes downstream cellular defects (Takano et al., 2017; Konig et al., 2019; Rosenwald et al., 2020). As the number of identified CI-inducing symbionts grows, it becomes increasingly important to establish testable models for the modification and rescue of these potentially diverse CI mechanisms.

## Data Availability Statement

The datasets presented in this study can be found in online repositories. The names of the repository/repositories and accession number(s) can be found below: Submitted to Dryad: https://doi.org/10.5061/dryad.4f4qrfj98.

## Author Contributions

MD designed and performed the experiments, analyzed the results, and wrote the first draft of the manuscript. SK helped take images on the confocal scope and generate wasps for experiments. SS-E designed FISH probes. SS-E and CS helped to optimize FISH assays for *Encarsia* testes. MH helped design to the experiments and analyze results. All authors contributed to the revision and editing of the manuscript. All authors contributed to the article and approved the submitted version.

## Conflict of Interest

The authors declare that the research was conducted in the absence of any commercial or financial relationships that could be construed as a potential conflict of interest.
