# Supplementary material for: Cardinium Localization During Its Parasitoid Wasp Host’s Development Provides Insights Into Cytoplasmic Incompatibility
Source: Front Microbiol. 2020 Dec 10;11:606399. doi: 10.3389/fmicb.2020.606399 (PMC7793848; doi:10.3389/fmicb.2020.606399)
Supplement: Supplementary file 1 [file Data_Sheet_1.docx]

**Supplemental methods – qPCR analysis of relative *Cardinium* density**

After male *E. suzannae* mated with females for CI crossing experiments, males were stored at -80°C until DNA extraction. Extractions consisted of homogenizing a male wasp in 3 µL of 20 mg ml ^-1^ proteinase k, then adding the homogenate to 50 µL of 5-10% w/v Chelex [1]. Samples were incubated at 37°C for 1 hour with periodic vortexing, followed by incubation at 97°C for 8 min and storage at -20°C. We estimated *Cardinium* density relative to host cells by performing quantitative PCR (qPCR) using Maxima SYBR Green/ROX qPCR Master Mix (2×) (ThermoFisher Scientific) with primers for the single-copy *Cardinium* *gyrB* gene [2] and the host *Ef1a* gene on a Bio-Rad CFX Connect Real-Time cycler [3]. We created standards via serial dilutions of PCR products from both primer sets. PCR products were diluted to a concentration of 1.0 ng/µL DNA, which was confirmed with a Qubit 4.0 fluorometer prior to serial dilution. Samples were run in triplicate and each qPCR plate included standards for both primer sets to correct for between-plate differences in reaction efficiency. Cycle quantification (C_q_) values were averaged and corrected before conversion to relative density, which was analyzed using the Mann-Whitney U-test in R 3.3.1 [4].

**Immunostaining of α-Tubulin**

To image the α-Tubulin (and complete sperm cell) composing the sperm tails in *E. suzannae*, we followed protocols modified from [5]. Testes from adult males were dissected in 1X PBS and placed in 4% paraformaldehyde fixative for 20 min. After fixation, testes were washed 3x in 1X PBT (1X PBS w/ 0.1% Triton-X) and allowed to rest in 1X PBT for 20 min. The testes were then incubated in 50 µL of the primary antibody, mouse anti-acetylated α-Tubulin (Santa Cruz Biotech; diluted 1:100 in 1X PBT), overnight at 4°C. The primary antibody was removed, and testes were washed 3X with 1X PBT. After washing, testes were incubated in 50 µL of the secondary antibody, anti-mouse Alexa488 (Santa Cruz Biotech; diluted 1:300 in 1X PBT), in the dark for 1 hr at room temperature. After incubation, testes were washing 3X with PBT and placed in 4% paraformaldehyde fixative for 40 min. This second fixation step prevents washing out of antibodies during downstream FISH reactions. After this second fixation step, testes were washed 3X in PBS and underwent FISH as described in the main text, starting with the serial ethanol dilution. After FISH, testes were stained with DAPI as described.

**Effects of cold exposure of sperm developmental schedule**

To investigate if cold exposure altered the timing of important stages of sperm development relative to pigmentation, a general developmental marker, we monitored developing male *E. suzannae* daily through the transparent whitefly cuticle and, when the late-stage ectoparasitic larvae deposited their meconium prior to the onset of pupation, they were removed from the leaf disk and placed individually into 1.2 ml vials plugged with cotton. Vials were then transferred to a Percival incubator set at either the control 27°C temperature or a cool 20°C day/ 17°C night treatment and monitored daily (16day:8night lightcycle). We collected male wasps during the red-eyed pupal stage, which at control temperatures contain testes with 1-2 elongating sperm cell cysts, and the fully melanized black pupal stage, in which these earliest developing sperm bundles have completed development and migrated into the seminal vesicle (sperm storage organ), to mark sperm development. We also recorded the developmental time for each pupal substage for control and cold-treated wasps.


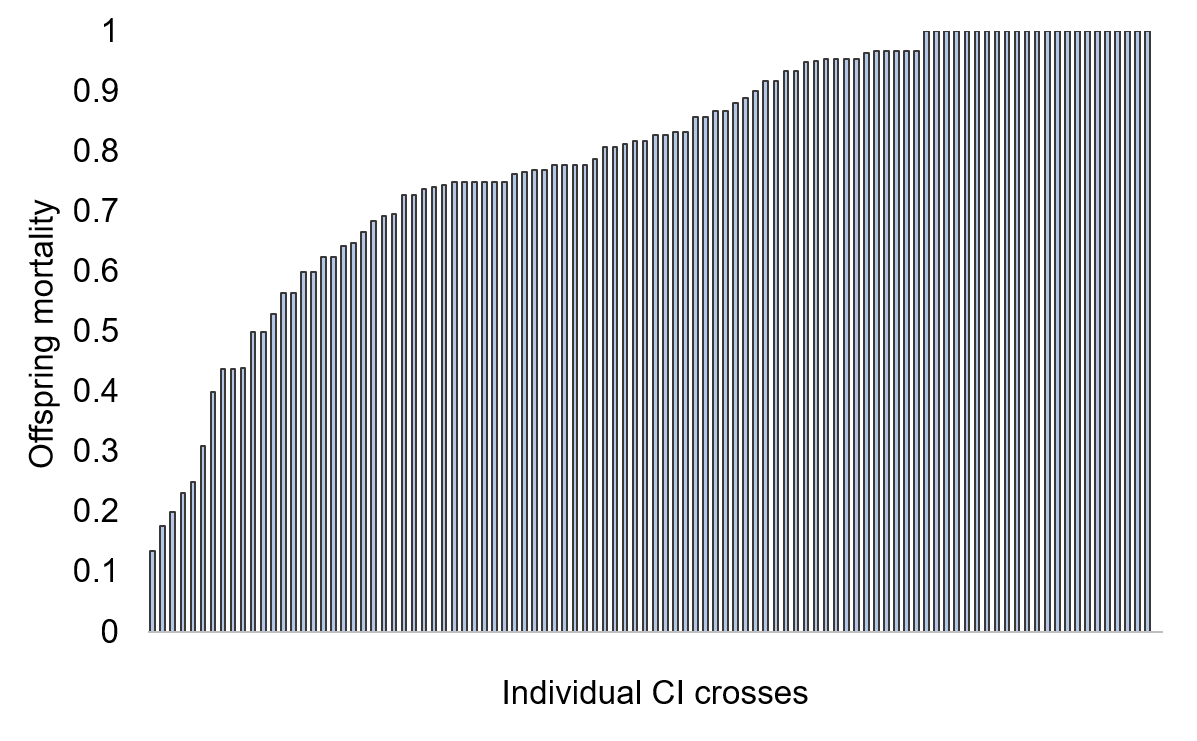


**SI Figure 1** Variation in levels of *Cardinium* CI-induced offspring mortality among individual crosses. Metadata compiled from CI crosses performed over 3 years, with each bar representing a CI cross with a single *Cardinium*-infected male. All crosses were performed under standard conditions (development at 27°C, 16:8h day/night cycles). Median = 0.82, 1^st^ quartile = 0.72, 3^rd^ quartile = 0.97.


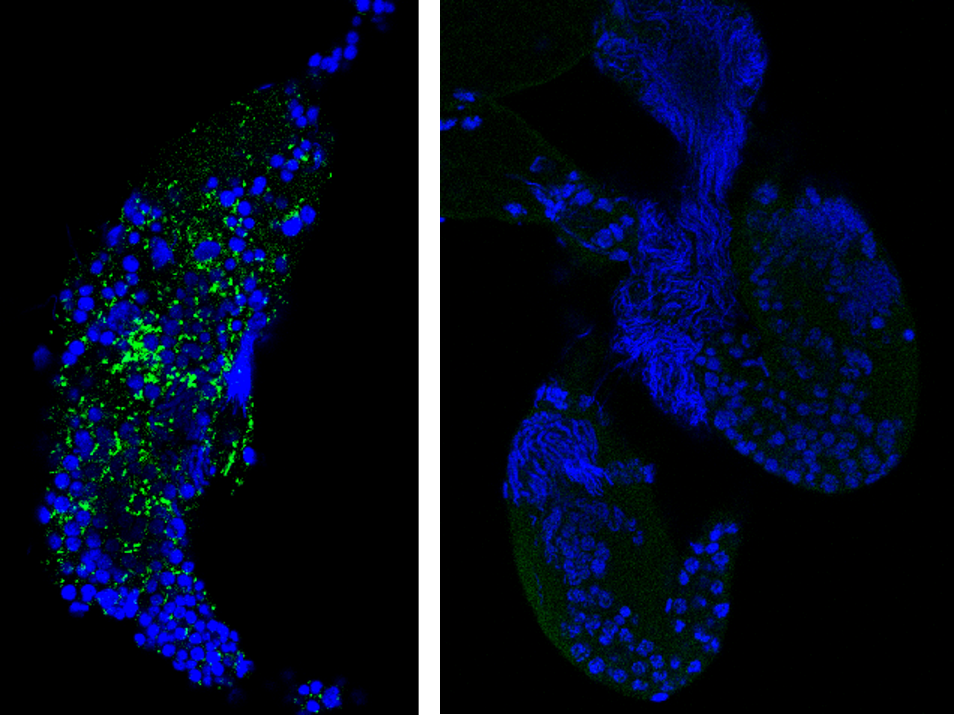


b.

a.

**SI Figure 2** Specificity of the *Cardinium* ch1162 16S rRNA fluorescent probe. FISH reactions were performed on testes from **a)** *Cardinium*-infected male *E. suzannae* and **b)** uninfected male *E. suzannae*. FISH reactions were performed on the same slide, with the same buffer source and reaction parameters. Blue = DAPI-stained host nuclei, green = ch1162 probe double-labeled with cy3.


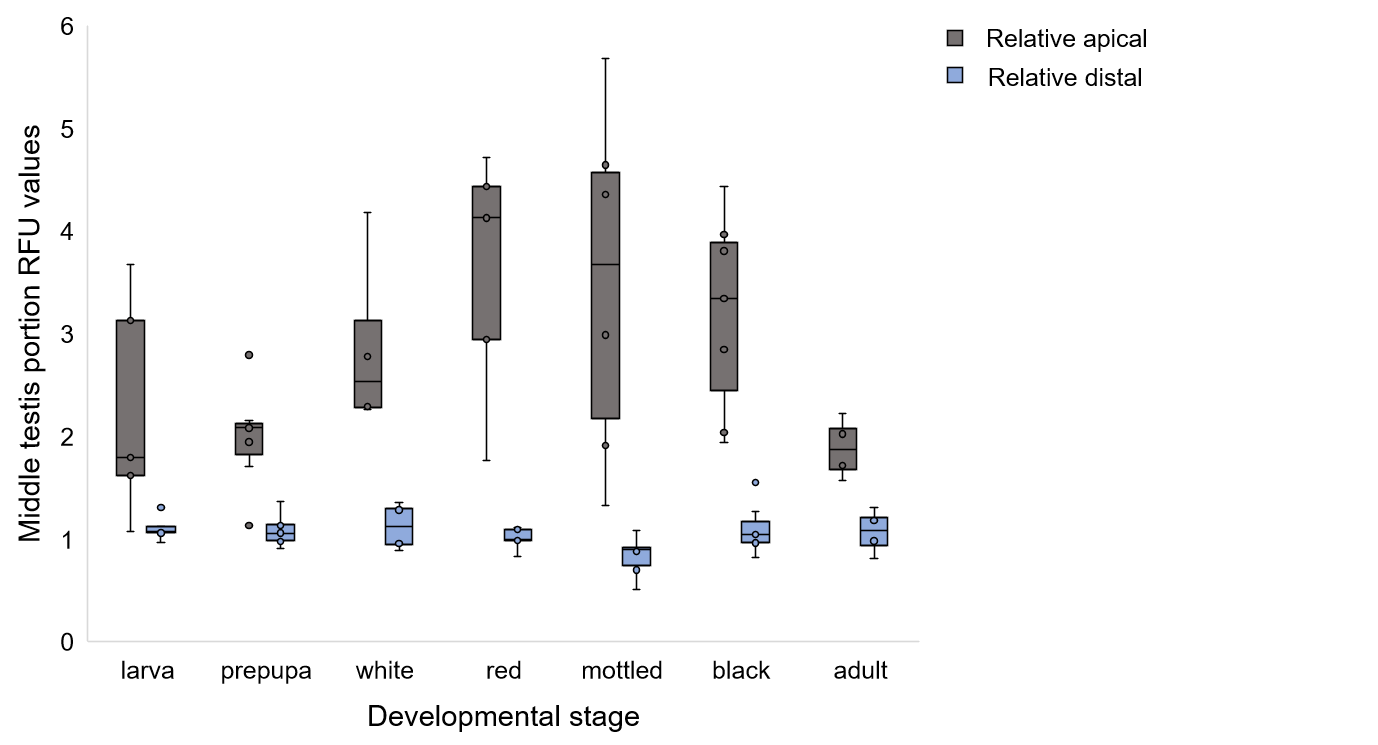


**SI Figure 3** *Cardinium* ch1162 probe Relative Fluorescence Units (RFU) values for the middle portion of the testis relative to the apical or distal regions. RFU values were calculated by averaging values of 3 cubic areas at three plane depths, for a total of 9 values per region in a sample in the Zen Black Software (Zeiss). The RFU values of *Cardinium* signal in the middle portion of the testis were approximately equal to that of the distal portion (blue) at all stages. The RFU values of *Cardinium* signal in the middle portion of the testis were consistently 2-3.5 times higher than the apical region, where *Cardinium* consistently appeared at low density. Sample size ranged from 4 – 7 testes per life stage.


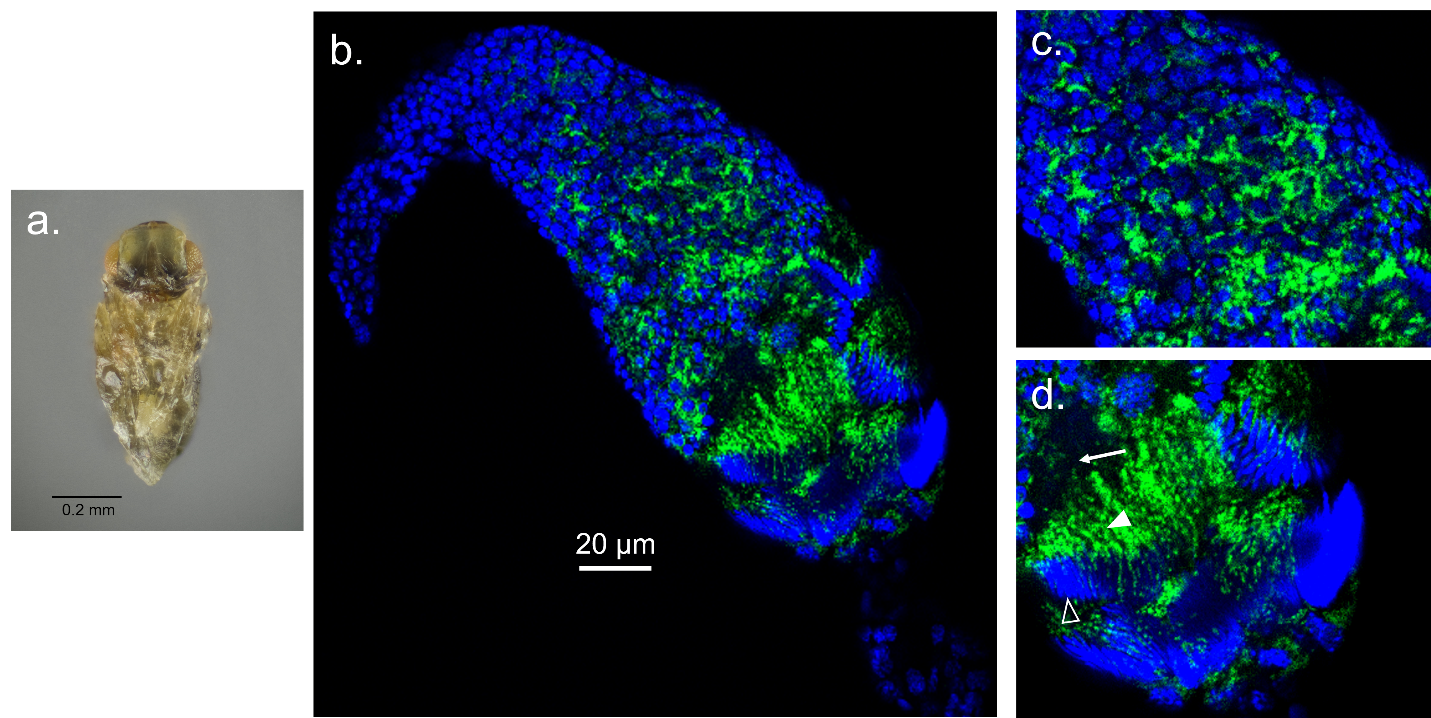


**SI Figure 4** Localization of *Cardinium* in the testes of mottled stage pupae. a) Mottled pupa showing light colored eye pigmentation and incomplete cuticular melanization. This stage occurs between the red-eyed and black pupal stage. b) Image of full testis, oriented from top left to bottom right, from the apical to the downstream reproductive tract. Note that the seminal vesicle (bottom right of b)), while present, does not contain mature sperm. c) Magnified mid region of testis, showing *Cardinium* cells co-localizing with developing spermatocytes. d) *Cardinium* is stripped away from sperm nuclei during the elongation period of spermiogenesis. The unfilled arrowhead and solid white arrowhead show elongating sperm nuclei and their resident *Cardinium* cells trailing behind, respectively. The white arrow refers to the extending sperm tails, appearing here as a void in the testis. Blue = DAPI-stained host nuclei, green = ch1162 probe double-labeled with cy3.


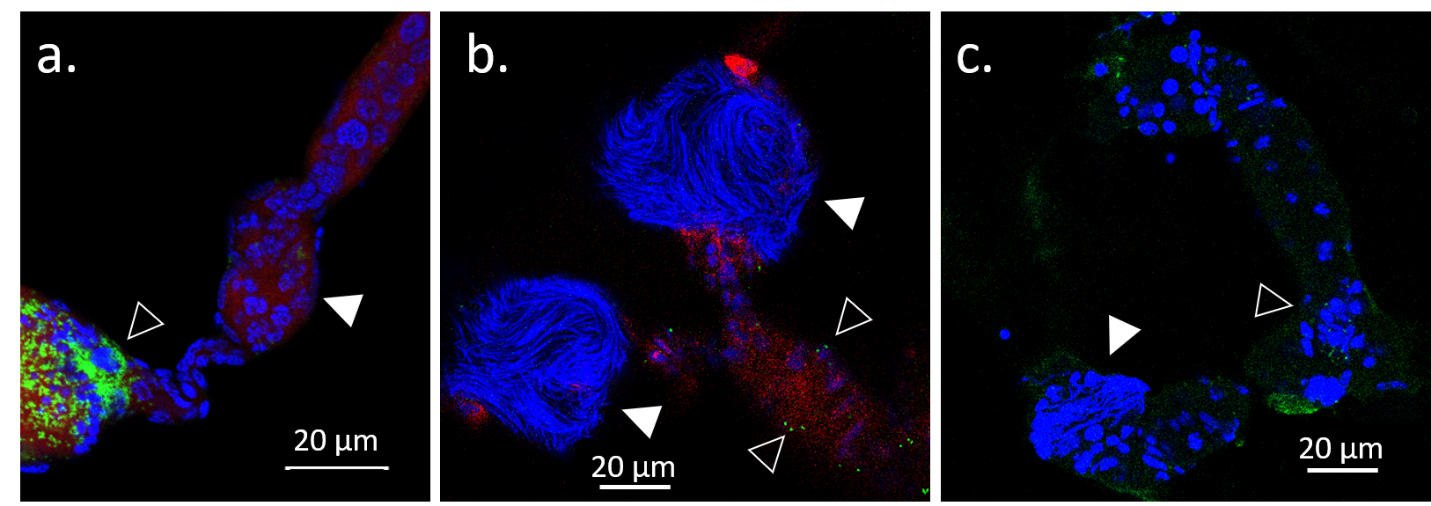


c.

b.

a.

**SI Figure 5** The seminal vesicle and downstream reproductive tract of *Encarsia* *suzannae*. **a)** Seminal vesicle (white arrowhead) of a red pupa that does not yet contain mature sperm. *Cardinium* cells (green) can be seen densely infecting the distal end of the testis leading to the seminal vesicle (unfilled arrowhead) **b)** Approximately 48hrs later, the seminal vesicles (white arrowheads) of a black pupal testis contain mature sperm but no *Cardinium* cells. Several *Cardinium* cells can be seen infecting the epithelial cells of the downstream reproductive tract (unfilled arrowheads) c) Seminal vesicle (white arrowhead) of a cold-exposed black pupa with *Cardinium* cells infecting host epithelial cells of the downstream reproductive tract (unfilled arrowhead). Blue = DAPI-stained host nuclei, green = ch1162 probe double-labeled with cy3, red in a, b = cytoplasmic probe labeled with cy5.

**SI Figure 6** *Encarsia suzannae* **a)** black pupal testis and **b**, **c)** mature sperm isolated from the seminal vesicle. The major protein in sperm tails, α-tubulin, is visualized with Alexa Fluor 488 using immunofluorescence following [5]. Green = α-tubulin, orange = *Cardinium* ch1162 (cy3), blue = DAPI stained host nuclei. *Cardinium* is absent from isolated sperm cells.

c.

b.

a.


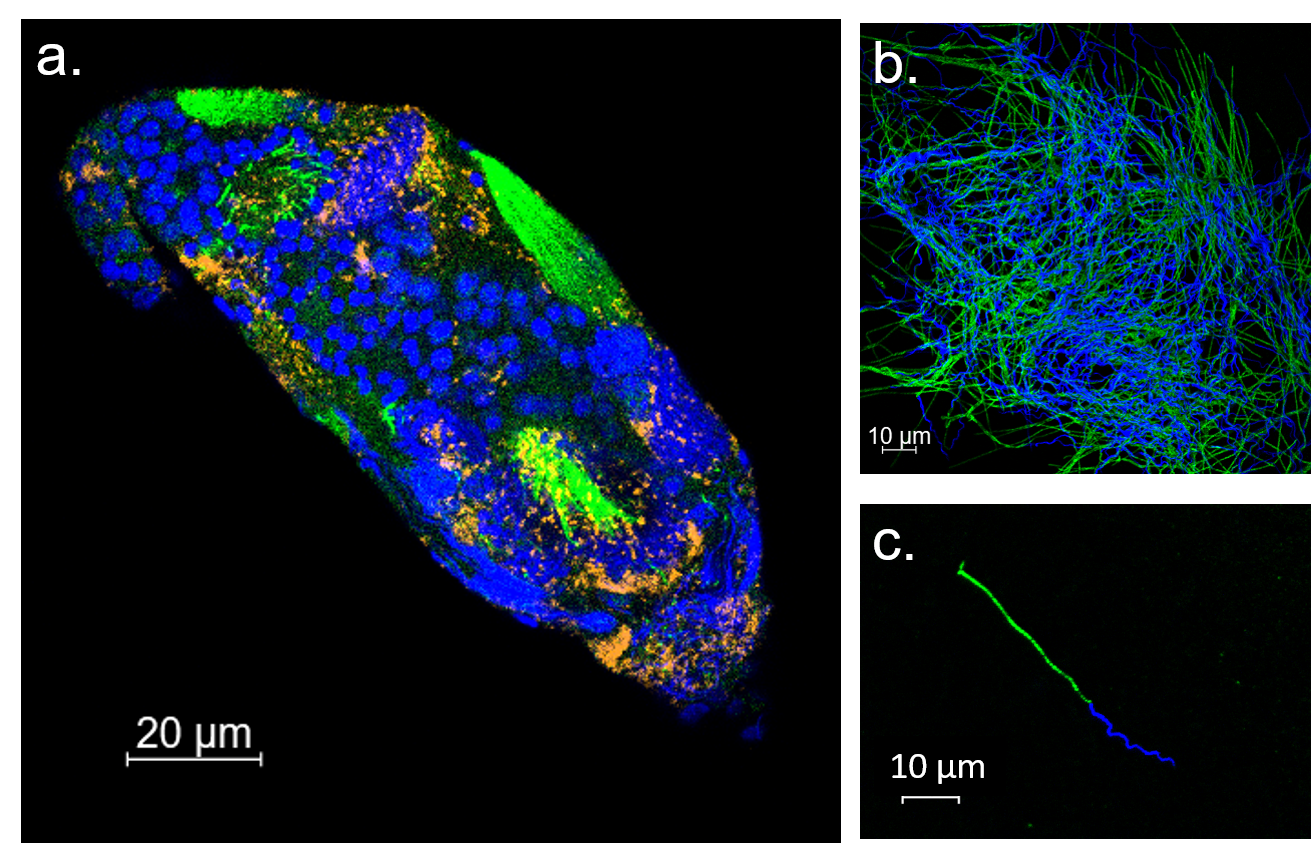


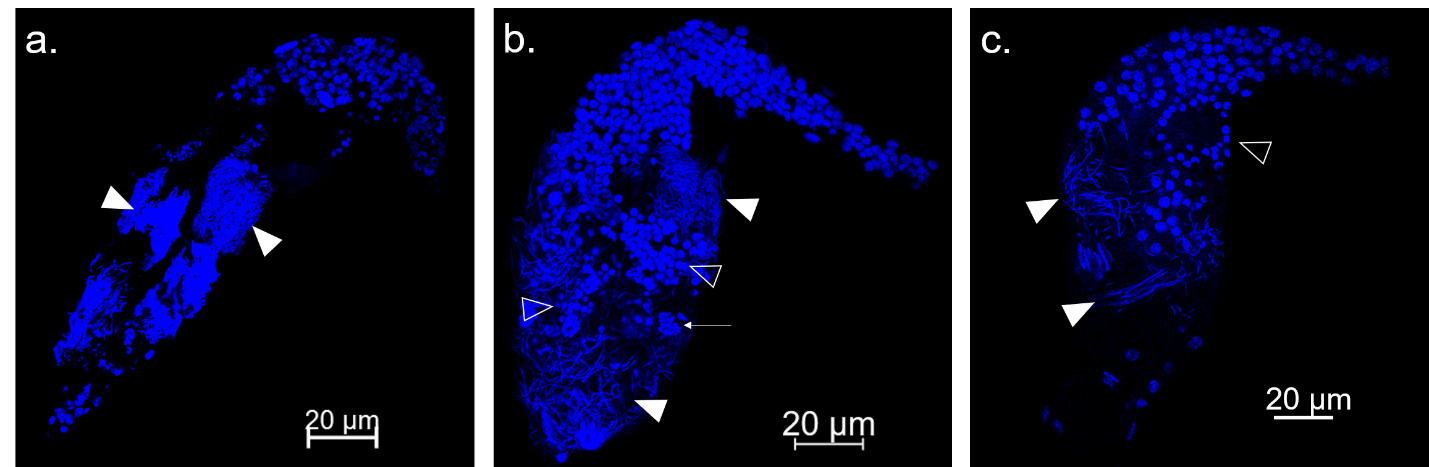


**SI Figure 7** *Encarsia suzannae* adult testes **a)** 48-hrs after emergence, **b)** 96-hrs after emergence, and **c)** 96-hrs and after mating with ~20 female wasps. Mature sperm are visible in all three testes (white arrowheads), although in b and c the sperm appear more disorganized. Some smaller nuclei, potentially belonging to developing spermatocytes, are present in **b)** (unfilled arrowheads). The larger nuclei of somatic cells (white arrow) can be more easily observed in later adult stages. Potential spermatocytes do not seem to mature further in response to mating in **c).** One cyst in **c)** is seen in the “cup” formation (clear arrowhead), indicating a low level of sperm development persists in the adult stage, possibly in response to mating. Blue = DAPI stained host nuclei.

c.

b.

a.

c.

b.


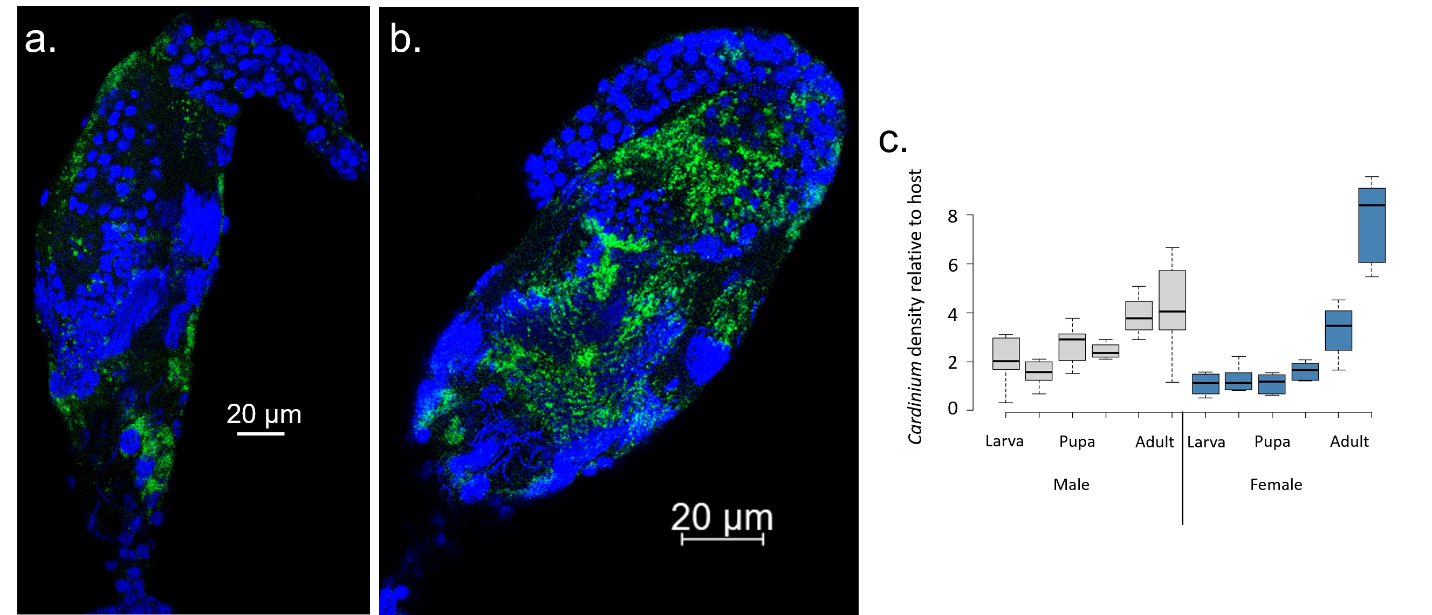


**SI Figure 8** *Cardinium* reaches variable densities in testes of different adult *Encarsia suzannae* **a)** testis with a lower density infection, **b)** testis with a high density *Cardinium* infection, and **c)** *Cardinium* density estimates (*gyrB* copy number) relative to whole wasp body (*ef1-α* copy number) using qPCR. qPCR estimates of *Cardinium* relative density are variable in the adult stage. Blue = DAPI stained host nuclei. Green = *Cardinium* ch1162 probe (cy3).

a.

b.


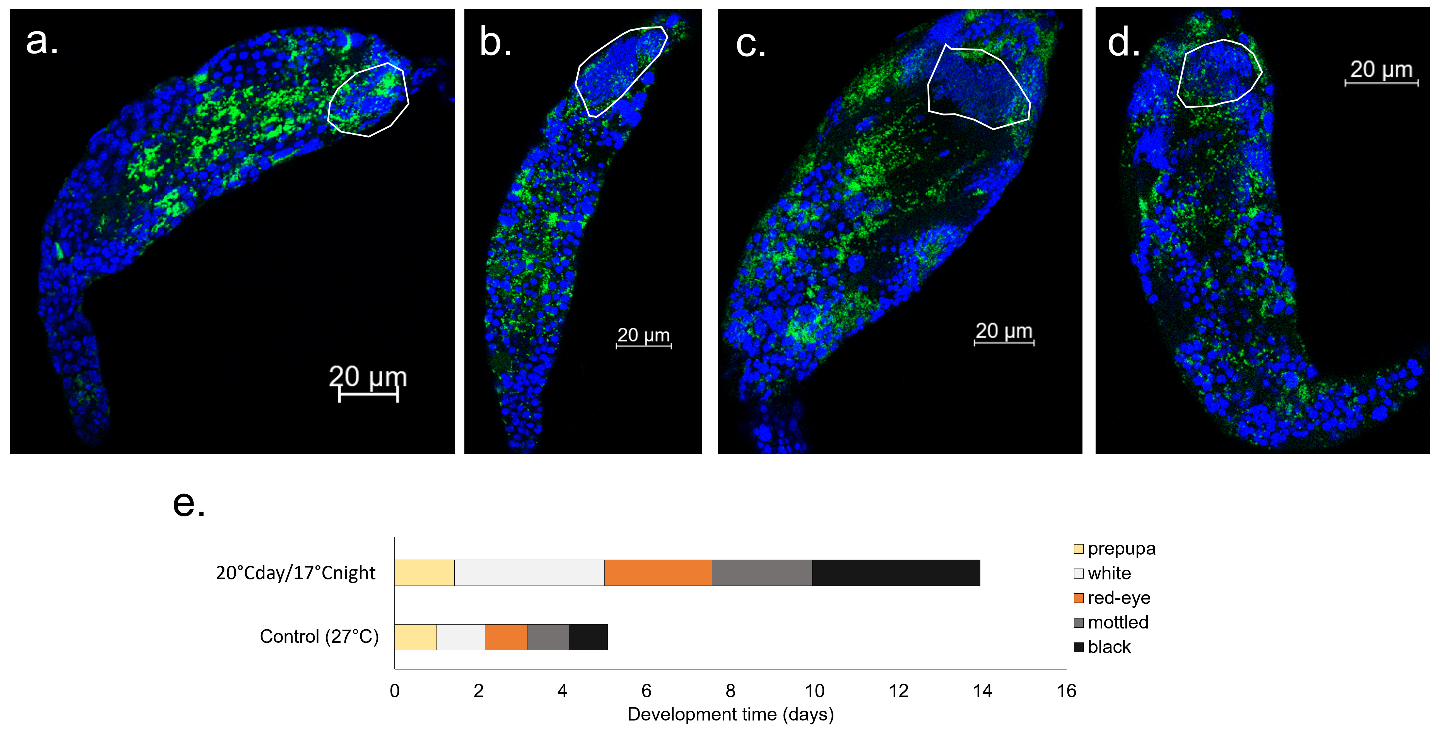


**SI Figure 8** Effects of cool temperatures on spermatogenesis. Testes of red-eyed pupal males **a)** raised at 27°C (control rearing temperature), and **b)** raised at cool 20°C/17°C treatment. Testes of black pupal males **c)** raised at 27°C (control rearing temperature), **d)** raised at a cool 20°C/17°C treatment. Circled regions for a-d refer to elongating nuclei of sperm cells undergoing spermiogenesis. The testes showed similar developmental schedules for the same stage regardless of temperature-induced delays. The green signal represents a *Cardinium* cy3-tagged 16S rRNA probe, and the blue signal represents a DAPI nuclear stain. **e)** The duration of pupal substages at control and cool temperatures in days.

References

[1] White, J.A., Kelly, S.E., Perlman, S.J. & Hunter, M.S. 2009 Cytoplasmic incompatibility in the parasitic wasp *Encarsia inaron*: disentangling the roles of *Cardinium* and *Wolbachia* symbionts. *Heredity* **102**, 483-489. (doi:10.1038/hdy.2009.5).

[2] Harris, L., Kelly, S., Hunter, M.S. & Perlman, S. 2010 Population dynamics and rapid spread of Cardinium, a bacterial endosymbiont causing cytoplasmic incompatibility in Encarsia pergandiella (Hymenoptera: Aphelinidae). *Heredity* **104**, 239-246.

[3] Stouthamer, C.M., Kelly, S. & Hunter, M.S. 2018 Enrichment of low-density symbiont DNA from minute insects. *Journal of microbiological methods* **151**, 16-19.

[4] R Core Team. 2016 R: A language and environment for statistical computing. Vienna, Austria, R Foundation for Statistical Computing.

[5] Ferree, P.M., Aldrich, J.C., Jing, X.Y.A., Norwood, C.T., Van Schaick, M.R., Cheema, M.S., Ausio, J. & Gowen, B.E. 2019 Spermatogenesis in haploid males of the jewel wasp *Nasonia vitripennis*. *Sci. Rep.* **9**. (doi:10.1038/s41598-019-48332-9).
